# Immunological and inflammatory profiles in mild and severe cases of COVID-19

**DOI:** 10.1038/s41467-020-17240-2

**Published:** 2020-07-08

**Authors:** Jin-Wen Song, Chao Zhang, Xing Fan, Fan-Ping Meng, Zhe Xu, Peng Xia, Wen-Jing Cao, Tao Yang, Xiao-Peng Dai, Si-Yu Wang, Ruo-Nan Xu, Tian-Jun Jiang, Wen-Gang Li, Da-Wei Zhang, Peng Zhao, Ming Shi, Chiara Agrati, Giuseppe Ippolito, Markus Maeurer, Alimuddin Zumla, Fu-Sheng Wang, Ji-Yuan Zhang

**Affiliations:** 1 Treatment and Research Center for Infectious Diseases, The Fifth Medical Center of PLA General Hospital, National Clinical Research Center for Infectious Diseases, 100039 Beijing, China; 2 Department of Clinical Medicine, Bengbu Medical College, 233000 Bengbu, China; 3 National Institute for Infectious Diseases Lazzaro Spallanzani-IRCCS- Via Portuense, 00179 Rome, Italy; 4 Immunotherapy Programme, Champalimaud Centre for the Unknown, 1400-038 Lisbon, Portugal; 5 I Med Clinic, University of Mainz, 55099 Mainz, Germany; 6 Department of Infection, Division of Infection and Immunity, University College London, London, UK; 7 National Institute for Health Research Biomedical Research Centre, University College London Hospitals NHS Foundation Trust, London, WC1E 6BT UK

**Keywords:** Infection, Viral infection, Acute inflammation

## Abstract

COVID-19 is associated with 5.1% mortality. Although the virological, epidemiological, clinical, and management outcome features of COVID-19 patients have been defined rapidly, the inflammatory and immune profiles require definition as they influence pathogenesis and clinical expression of COVID-19. Here we show lymphopenia, selective loss of CD4+ T cells, CD8+ T cells and NK cells, excessive T-cell activation and high expression of T-cell inhibitory molecules are more prominent in severe cases than in those with mild disease. CD8+ T cells in patients with severe disease express high levels of cytotoxic molecules. Histochemical studies of lung tissue from one fatality show sub-anatomical distributions of SARS-CoV-2 RNA and massive infiltration of T cells and macrophages. Thus, aberrant activation and dysregulation of CD8+ T cells occur in patients with severe COVID-19 disease, an effect that might be for pathogenesis of SARS-CoV-2 infection and indicate that immune-based targets for therapeutic interventions constitute a promising treatment for severe COVID-19 patients.

## Abstract (web summary)

Immunophenotyping of patients with COVID-19 is ongoing, but much remains to be learned. Here the authors analyze 41 hospitalized patients with COVID-19 and show a higher degree of lymphopenia in various immune cell subsets as well as cytotoxicity and T cell inhibitory marker expression in severe cases compared with mild.

## Introduction

As of 26th June 2020, the unprecedented global epidemic outbreak of novel Coronavirus Disease-2019 (COVID-19) has resulted in 9,296,202 laboratory-confirmed cases of SARS-CoV-2 infection reported from 210 countries to the WHO^1^. Of these, there have been 479,133 deaths (5.1% mortality). Whilst the virological, epidemiological, clinical, routine laboratory, imaging, and management outcome features of COVID-19 patients have been rapidly defined^2–13^, the immune and inflammatory profiles are not well understood.

A wide spectrum of clinical manifestations occurs from mild to severe disease with extensive lung involvement leading to acute respiratory distress syndrome (ARDS) and death^2–7,10,13^. Severe lung disease with extensive alveolar damage and progressive respiratory failure leads to fatal outcomes^3–5,7,11^. Higher fatality rates occur in the elderly, and in individuals who have diabetes, co-morbidities and other causes of immunocompromise^4^. We recently reported a case where significant interstitial lymphocytic infiltrates in the lung tissue, lymphopenia, and overactivation of T cells in peripheral blood^10^. Inflammatory and immune responses are important for elimination of the infection, but may have a significant impact on SARS-CoV-2 pathogenesis, and may play a role in the expression of the clinical spectrum of COVID-19 disease.

In this study, we perform a cross-sectional observational study of immunological and inflammatory markers and cells in 41 COVID-19 patients (29 with mild disease and 12 with severe disease including 2 fatal cases). We find COVID-19 patients in severe group are characterized by profound lymphopenia, strong T-cell activation, and increased expression of T-cell inhibitory molecules compared to mild group of patients.

## Results

### Patient demographics and clinical features

Forty-one patients with laboratory-confirmed acute COVID-19 disease were enrolled: 29 with mild disease and 12 with severe disease. Representative computed tomographic images are shown in Supplementary Fig. 1. Table 1 shows demographics and baseline characteristics of these patients. The median age was 39 years (IQR 33.5–50.0); 25 (61%) were male. The median time of symptom onset before admission was 5 days (IQR 4.0–9.0). Two patients (5%) died. Thirty-six patients (85%) reported fever before hospitalization. Signs and symptoms included myalgia or fatigue (46%), expectoration (32%), cough (27%), sore throat (17%), diarrhea (10%), shortness of breath (12%), rhinorrhea (2%), and chest pain (2%). The median age was significantly higher in severe group (50 years) compared to mild group (37 years, *p* = 0.0072, two-tailed Mann–Whitney *U*-test). The duration of symptoms before admission was also higher in severe group (10.5 vs. 5.0 days, *p* = 0.0064, two-tailed Mann–Whitney *U*-test). Symptoms such as expectoration, myalgia or fatigue, and shortness of breath were increased in severe group compared to mild group.

**Table 1:** Demographics and baseline characteristics of patients infected with SARS-CoV-2.

	Total (*n* = 41)	Mild (*n* = 29)	Severe (*n* = 12)	*p* value
Age, years	39.0 (33.5–50.0)	37.0 (29.0–44.0)	50.0 (39.75–85.0)	0.0072
Sex				0.6308
Men	25 (61%)	17 (59%)	8 (67%)	
Women	16 (39%)	12 (41%)	4 (33%)	
OnSet of symptom to hospital admission, days	5.0 (4.0–9.0)	5.0 (4.0–7.0)	10.5 (5.0–12.25)	0.0064
Death	2 (5%)	0	2 (17%)	0.0805
Exposure to Wuhan	24 (59%)	18 (62%)	6 (50%)	0.4754
Any comorbidity	10 (24%)	3 (10%)	7 (58%)	0.0011
Hypertension	5 (12%)	1 (3%)	4 (33%)	0.0078
Diabetes	4 (10%)	2 (7%)	2 (17%)	0.3374
Malignancy	1 (1%)	0	1 (8%)	0.2927
Chronic liver disease	3 (7%)	1 (3%)	2 (17%)	0.2002
Fever	35 (85%)	25 (86%)	10 (83%)	0.8128
Highest temperature (°C)				0.63
<37.3	6 (15%)	4 (14%)	2 (17%)	
37.3–38.0	9 (22%)	7 (24%)	1 (8%)	
38.1–39.0	18 (44%)	12 (41%)	7 (58%)	
>39.0	8 (20%)	6 (21%)	2 (17%)	
Cough	27 (66%)	17 (59%)	10 (83%)	0.1289
Expectoration	13 (32%)	6 (21%)	7 (58%)	0.0184
Rhinorrhoea	1 (2%)	1 (3%)	0	>0.9999
Myalgia or fatigue	19 (46%)	9 (31%)	10 (83%)	0.0022
Nausea and vomiting	0	0	0	
Sore throat	7 (17%)	5 (17%)	2 (17%)	0.9645
Shortness of breath	5 (12%)	1 (3%)	4 (33%)	0.0078
Chest pain	1 (2%)	0	1 (8%)	0.2927
Diarrhea	4 (10%)	4 (14%)	0	0.1756

Data are median (IQR), or *n* (%). *p* values comparing mild and severe are from two-tailed *χ*² test, two-tailed Fisher’s exact test, or two-tailed Mann–Whitney *U*-test.

### Laboratory findings

Routine laboratory test results are shown in Table 2. There were no significant differences in the absolute number of leukocytes, neutrophils, and platelets counts between mild and severe group. The severe group exhibited a significantly lower absolute lymphocyte count than the mild group (*p* < 0.0001, two-tailed Mann–Whitney *U*-test). The severe group showed increased D-dimer (*p* = 0.0065, two-tailed Mann–Whitney *U*-test) and serum ferritin (*p* = 0.0024), and had a lower albumin levels (*p* = 0.0005, two-tailed Mann–Whitney *U*-test).

**Table 2:** Laboratory findings of hospitalized patients infected with SARS-CoV-2.

	Normal range	Total (*n* = 41)	Mild (*n* = 29)	Severe (*n* = 12)	*p* value
Leukocytes count (×10^9^ L^−1^)	3.97–9.15	4.95 (4.02–6.68)	4.95 (4.02–6.35)	4.955 (3.9975–6.695)	0.9718
Neutrophils count (×10^9^ L^−1^)	2.0–7.0	2.93 (2.25–4.36)	2.85 (2.24–3.75)	3.715 (2.7425–5.04)	0.2136
Lymphocytes count (×10^9^ L^−1^)	0.80–4.00	1.31 (0.88–1.61)	1.54 (1.17–1.82)	0.775 (0.4925–1.0425)	<0.0001
Platelets count (×10^9^ L^−1^)	85.0–303.0	170.0 (151.0–233.0)	170.0 (151.0–218.0)	170.0 (149.0–263.5)	0.5854
Hemoglobin (g L^−1^)	131.0–172.0	136.0 (128.0–148.0)	140.0 (130.0–150.0)	127.0 (113.5–147.25)	0.114
Activated partial thromboplastin time (s)	23.0–42.0	30.4 (27.4–34.2)	30.4 (28–34.7)	30.1 (24.8–32.025)	0.3382
Prothrombin time (s)	10.20–14.30	11.9 (11.4–12.5)	12.1 (11.5–12.5)	11.65 (11.325–11.95)	0.0922
D-dimer (µg L^−1^)	0.00–0.55	0.24 (0.17–0.53)	0.23 (0.17–0.42)	0.61 (0.245–0.985)	0.0065
Albumin (g L^−1^)	35.0–55.0	39.0 (35.0–42.0)	41.0 (38.0–43.0)	34.5 (32.5–36.25)	0.0005
Alanine aminotransferase (U L^−1^)	5.0–40.0	26.0 (15.0–40.0)	24.0 (15.0–39.0)	26.0 (14.75–70.75)	0.6456
Aspartate aminotransferase (U L^−1^)	8.0–40.0	24.0 (19.0–35.0)	25.0 (21.0–30.0)	21.5 (18.0–55.0)	0.5756
Total bilirubin (μmol L^−1^)	3.40–20.5	10.3 (7.3–14.7)	9.5 (7.2–14.7)	10.95 (8.825–14.2)	0.5104
Serum creatinine (μmol L^−1^)	62.0–115.0	74.0 (69.0–85.0)	80.0 (69.0–87.0)	70.0 (67.0–79.0)	0.0355
Lactate dehydrogenase (U L^−1^)	109.0–245.0	212 (193–247)	212 (193–247)	210.5 (190.5–333.25)	0.7185
Interleukin-6 (pg mL^−1^)	0.0–7.0	9.335 (5.383–23.8775)	8.545 (5.395–16.865)	18.695 (3.84–40.8175)	0.6556
C-reactive protein (mg L^−1^)	0.068–8.20	7.4 (4.0–16.1)	7.1 (4.0–10.4)	10.955 (5.4125–36.55)	0.1602
Procalcitonin (ng mL^−1^)	0.0–0.5	0.044 (0.035–0.065)	0.0435 (0.034–0.0615)	0.049 (0.0385–0.073)	0.4569
Erythrocyte sedimentation rate (mm h^−1^)	0.0–15.0	12.5 (7.75–34.25)	11.5 (7–19.75)	26.5 (11.5–61)	0.0699
Serum ferritin (ng mL^−1^)	30.0–400.0	366.95 (187.5–538.025)	306.8 (95.32–434.9)	532 (419.7–837.6)	0.0024

Data are median (IQR). *p* values comparing mild and severe are from two-tailed Mann–Whitney *U*-test.

### T cells, B cells, and NK cells profiles

**Fig. 1:**
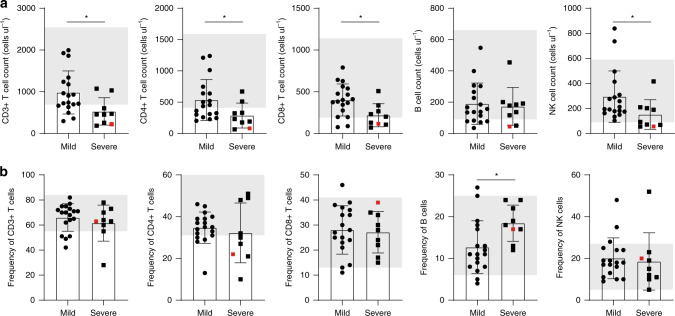
Absolute cell counts and percentages of peripheral lymphocyte subsets in SARS-CoV-2 infected patients. **a** Absolute cell counts and **b** percentages of CD3+ T cells, CD4+ T cells, CD8+ T cells, B cells, and NK cells in the mild (*n* = 18) and severe (*n* = 9) group were analyzed by flow cytometry. Shaded region showing the normal range of indicated cell subset. Colored symbols indicated deceased cases. Data are expressed as mean ± SD. *p < 0.05 by two-tailed Mann–Whitney *U*-test for **a** (*p* = 0.0124, *p* = 0.0354, *p* = 0.0199, and *p* = 0.0354, respectively) and **b** (*p* = 0.015). Source data included as a Source Data File.

The absolute numbers of CD3+ T cells, CD4+ T cells, CD8+ T cells, B cells, and NK cells are shown in Fig. 1a. The severe group had decreased numbers of CD3+ T cells, CD4+ T cells, CD8+ T cells, and NK cells compared to the group experienced a mild clinical presentation. 7 out of 9 (77.8%) patients exhibited decreased CD3+ T cell and CD4+ T cell counts, and 4 out of 9 (44.4%) patients had decreased CD8+ T cell and NK cell counts below normal levels. However, in the patient group with mild symptoms, 5 out of 18 (27.8%) patients exhibited decreased CD3+ T cell counts, 8 out of 18 (44.4%) patients exhibited decreased CD4+ T cell count. 2 out of 18 (11.1%) patients had decreased CD8+ T cell counts. NK cell count was in normal range for most mild patient (16/18), with two patients exhibited higher NK cells than normal. No statistical differences were observed in the frequencies of CD3+ T cells, CD4+ T cells, CD8+ T cells, and NK cells between mild and severe groups, except for that of B cells, which was increased in severe patients (*p* = 0.015, two-tailed Mann–Whitney *U*-test) (Fig. 1b).

### CD8+ T cells in severe patients

**Fig. 2:**
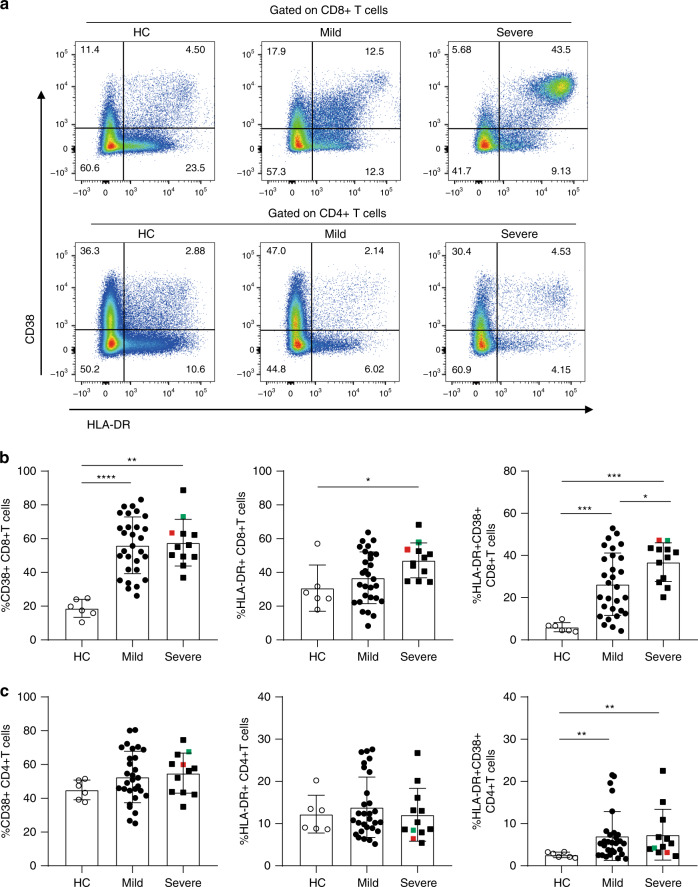
Increased expression of activation markers on T cells in SARS-CoV-2 infected patients. **a** Representative dot plots showing CD38 and HLA-DR expression on CD8+ and CD4+ T cells from PBMCs of HC, Mild and Severe COVID-19 patients. **b**, **c** The percentage of CD38 and HLA-DR single positive or double positive **b** CD8+ T cells or **c** CD4+ T cells in PBMCs of HC (*n* = 6), mild (n = 29) and severe (*n* = 12) SARS-COV-2 infected patients was assessed by flow cytometry. Colored symbols indicated deceased cases. Data are expressed as mean ± SD. *p < 0.05, ***p < 0.001, ****p < 0.0001, by two-tailed Mann–Whitney *U*-test for **b** (*p* < 0.0001 and *p* = 0.0001 for CD38+CD8+T cells, *p* = 0.0135 for HLA-DR+CD8+T cells, and *p* = 0.0002, *p* = 0.0001, and *p* = 0.0372 for HLA-DR+CD38+CD8+T cells) and **c** (*p* = 0.0088 and *p* = 0.0046 for HLA-DR+CD38+CD4+T cells, respectively). Source data included as a Source Data File.

T-cell activation plays a critical role in defense of pathogen infection. Thus, we evaluated the degree of T-cell activation. The levels of T-cell activation were statistically higher in SARS-CoV-2 infected patients than cells from healthy controls (HC), as reflected by higher proportions of CD38+CD8+T cells, HLA-DR+CD8+T cells, and CD38+HLA-DR+CD8+T cells (Fig. 2a, b). Notably, the frequencies of CD38+HLA-DR+CD8+T cells in patients with severe symptoms were significantly higher compared with patients in the mild symptom group (*p* = 0.0372, two-tailed Mann–Whitney *U*-test) (Fig. 2b). The frequencies of CD38+HLA-DR+CD8+T and HLA-DR+CD8+T cells correlated positively with time after disease onset (*R* = 0.4425, *p* = 0.0038 and *R* = 0.4300, *p* = 0.005, respectively, Pearson correlation test) (Supplementary Fig. 2a). For CD4+T cells, no statistically differences were observed for frequencies of CD38+CD4+T cells and HLA-DR+CD4+T cells among HC, mild, and severe patients. However, compared with HC, the proportion of CD38+HLA-DR+CD4+T cells was higher in both mild and severe patients, but without difference between the two groups of SACR-CoV-2 infected patients (Fig. 2c). There is no difference in the absolute number of CD38+HLA-DR+CD8+T and CD38+HLA-DR+CD4+T cells between mild and severe group (Supplementary Fig. 2b).

### PD-1 and TIM-3 expression on CD8+ T cells in severe patients

**Fig. 3:**
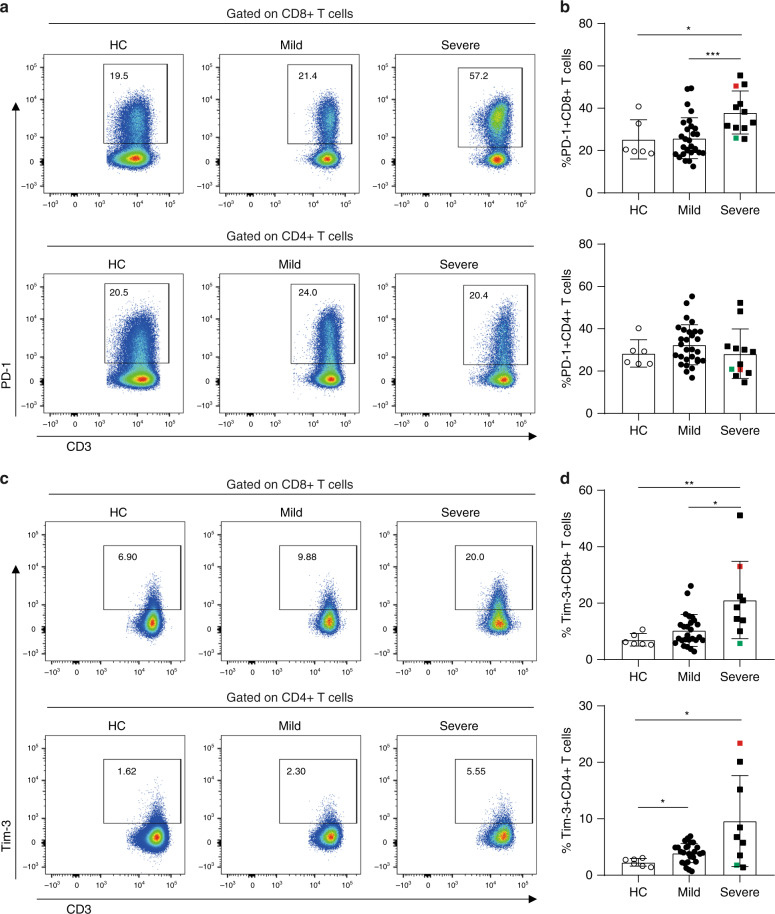
Exhaustion of T cells in SARS-COV-2 infected patients. **a**, **b** PD-1 expression on CD8+ T and CD4+ T cells in HC (*n* = 6), mild (*n* = 29), and severe (*n* = 12) patients of SARS-COV-2 infection. **c**, **d** TIM-3 expression on CD8+ T and CD4+ T cells in HC (*n* = 6), mild (*n* = 29), and severe (*n* = 12) patients of SARS-COV-2 infection. Colored symbols indicated deceased cases. Data are expressed as mean ± SD. *p < 0.05, **p < 0.01, ***p < 0.001, by two-tailed Mann–Whitney *U*-test for **b** (*p* = 0.032 and *p* = 0.0005) and **d** (*p* = 0.0076 and *p* = 0.0105 for Tim-3+CD8+ T cells, and *p* = 0.0185 and *p* = 0.0496 for Tim-3+CD4+ T cells, respectively). Source data included as a Source Data File.

The activation/inhibitory costimulatory molecules expression on circulating T cells is shown in Fig. 3. The proportion of PD-1+CD8+T cells was higher in severe patients compared to HC and mild patients, but there was no statistical difference in the proportion of PD-1+CD4+T cells among HC, mild and severe patients (Fig. 3a, b). In addition, the frequencies of PD-1+CD8+T cells correlated positively (*R* = 0.4271, *p* = 0.0053, Pearson correlation test) with time after disease onset (Supplementary Fig. 2c). Severe patients also exhibited higher frequencies of TIM-3+CD8+T cells compared to HC and mild patients. Increased frequencies of TIM-3+CD4+T cells were also observed in mild and severe patients compared with HC. However, no statistically significant difference was observed in the frequencies of TIM-3+CD4+T cells between mild and severe patients (Fig. 3c, d). Interestingly, positive correlations were observed between the frequencies of Tim-3+CD8+T (*R* = 0.5468, *p* = 0.0007, Pearson correlation test) and Tim-3+CD4+T cells (*R* = 0.36, *p* = 0.0337, Pearson correlation test) and time after disease onset (Supplementary Fig. 2d). In addition, the frequencies of PD-1 on CD38+HLA-DR+CD8+T and CD38+HLA-DR+CD4+T cells were higher than their double negative counterparts, respectively. Compared with mild cases, PD-1 expression on CD38+HLA-DR+CD4+T and CD38+HLA-DR+CD8+T cells are higher in severe cases (Supplementary Fig. 2e).

### CD4+ T cells differentiation

The naïve/memory subsets of CD4+ T cells were analyzed according to CD45RA, CD27, and CCR7 expression (Supplementary Fig. 3a). Compared with HC, SARS-COV-2 infection leads to increased proportion of *T*_N_ cells in severe patients and increased *T*_CM_ cells in mild patients. Decreased proportion of *T*_TM_ cells was observed in mild patients compared with HC. No statistical differences were observed between mild and severe group in the proportions of memory subsets.

The functional polarization of CD4+ T cells was defined according to the chemokine receptors CCR4, CCR6, and CXCR3 expression (Supplementary Fig. 3b). Compared with HC, the percentages of Th1 and Th1Th17 cells were higher in mild patients, while the percentages of Th2 and Th17 cells were lower. No statistical differences were observed in the percentages of Th1, Th2, and Th17 cells between mild and severe patients. The frequencies of Treg and pTfh cells were also assessed, but there was no statistical difference among HC, mild and severe groups (Supplementary Fig. 3c, d).

### Cytokines and chemokines

Cytokines and chemokines in plasma of COVID-19 patients and HC were measured using flow cytometry based Aimplex kit (Supplementary Fig. 4). Inflammatory cytokines and chemokines in SARS-CoV-2 infected patients, such as IL-6, TNF-α, IL-17A, IFN-γ, and MCP-1, were significantly elevated than HC, although there was no significance between mild and severe patients. Cytokines related tissue repair, such as IL-10, IL-4, and IL-5, were also upregulated in COVID-19 patients, but without statistical significance between mild and severe patients. In summary, similar to SARS-CoV^14^ and MERS-CoV^15^ infections, COVID-19 patients showed higher plasma concentrations of cytokines/chemokines than HC, albeit there was no difference between mild and severe cases.

### Multifunctional cytotoxic CD8+ T cells

**Fig. 4:**
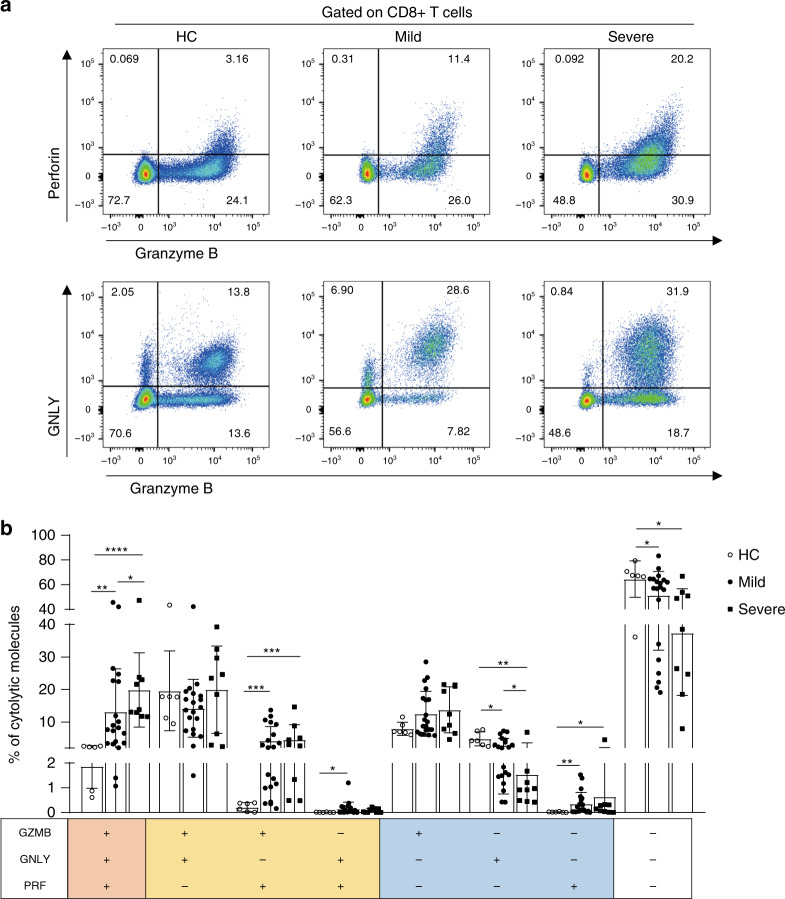
Co-expression patterns of cytotoxic effectors in CD8+ T cells from SARS-COV-2 infected patients. **a**, **b** Cytolytic molecules were assessed by flow cytometry, and the proportions of CD8+ T lymphocytes co-expression of GNLY, GZMB, PRF were analyzed in HC (*n* = 6), mild (*n* = 20), and severe (*n* = 9) patients of SARS-COV-2 infection. Data are expressed as mean ± SD. *p < 0.05, **p < 0.01, ***p < 0.001, ****p < 0.0001 by two-tailed Mann–Whitney *U*-test for **b** (*p* = 0.002, *p* = 0.0004, and *p* = 0.0386 for GZMB+GNLY+PRF+ cells, *p* = 0.0006 and *p* = 0.0004 for GZMB+GNLY-PRF+ cells, *p* = 0.0459 for GZMB-GNLY+PRF+ cells, *p* = 0.0331, *p* = 0.0048, and *p* = 0.03 for GZMB-GNLY+PRF− cells, *p* = 0.0026 and *p* = 0.0496 for GZMB-GNLY-PRF+ cells, and *p* = 0.0279 and *p* = 0.012 for GZBM-GNLY-PRF− cells, respectively). Source data included as a Source Data File.

Phenotypes of circulating CD8+ T cells were analyzed (Supplementary Fig. 5a). Among the CD8+ T cells, the frequencies of *T*_TM_ subset significantly decreased in SARS-CoV-2 infected patients, but without difference between mild and severe individuals (Supplementary Fig. 5b). Moreover, in severe patients, there was an increase of *T*_E_ subset compared to HC and a decrease of *T*_CM_ subset compared to mild patients (Supplementary Fig. 5b). No significant changes were seen in *T*_N_ or *T*_EM_ subsets. The expressional levels of cytotoxic molecules (granzyme B, GZMB; perforin, PRF and granulysin, GNLY) in CD8+ T cells were analysed. Based on cytolytic ability, CD8+ T cells were divided into four subpopulations: monocytotoxic T lymphocytes (M-CTL) expressing only one of GZMB, PRF, and GNLY; dicytotoxic T lymphocytes (D-CTL) expressing two kinds of cytotoxic molecules; tricytotoxic T lymphocytes (T-CTL) expressing all three cytotoxic molecules; and noncytotoxic T lymphocytes (N-CTL) which express none of the molecules. The data showed that the frequencies of T-CTL are the highest in CD8+ T cells from severe patients and the lowest in HCs (Fig. 4a, b). There were also higher expressional levels of GZMB and PRF double positive D-CTL in SARS-CoV-2 infected patients compared to HCs (Fig. 4b). The high cytotoxicity of CD8+ T cells coincides with their over-activated status in severe patients, which might contribute to the ongoing immune injury in lung tissue.

### Immunobiological findings

**Fig. 5:**
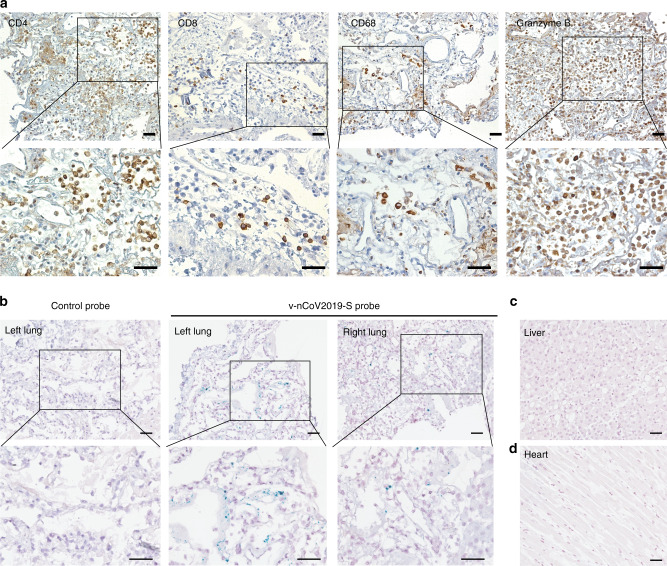
Lymphocyte infiltration and viral distribution in lung. **a** Immunohistochemical staining with anti-CD4, anti-CD8, anti-CD68 and anti-GZMB in lung tissues, scale bar = 50 μm. **b**–**d** Representative microscopy images of both left and right lungs (**b**), liver (**c**), and heart (**d**) sections from a COVID-19 patient hybridized with control or SARS-CoV-2-specific probes, scale bar = 50 μm. Independent experiments are repeated at least three times.

Pulmonary infiltration of lymphocytes and viral distribution in tissues are shown in Fig. 5a–d. There was diffuse alveolar damage with cellular fibromyxoid exudates and desquamation of pneumocytes in both lungs. Massive CD4+ T cell, CD8+ T cell, macrophages and GZMB+ cells infiltration were observed in the interstitial area of lung tissues (Fig. 5a). We used an in situ hybridization assay to detect viral RNA in fixed tissue sections. Intense dot signals were observed both in the left and right lungs (Fig. 5b), suggesting active replication in these cells. Indeed, these cells with intense signals showed atypical enlarged pneumocytes nuclei and prominent nucleoli, which are typical characteristics for multinucleated syncytial cells. Punctate dots were mainly distributed in the intraalveolar spaces, which might be dissociative viral particles. Notably, viral signal was not observed in liver (Fig. 5c) or heart tissues (Fig. 5d).

## Discussion

To our knowledge this is the first detailed study of the immunological and inflammatory profiles of COVID-19 patients with severe disease. Cellular immune responses, consisting of both CD4+ T and CD8+ T cells, are essential for the control of viral infection. Much work has been aimed to elucidate the virological features^3,6,9,11–13,16–19^ as well as clinical characteristics^3–5,7,16,17^ of COVID-19 patients. However, little is known about the immunological and inflammatory profiles of patients and their association with COVID-19 severity.

The clinical, imaging, and routine laboratory findings of enrolled patients are similar to recently published data^2–7,10,11,13^. Lymphocytopenia has been observed in COVID-19 patients showing different spectrum of clinical disease^20^, although the changes in different lymphoid subsets were not yet defined. In a previous study, mainly in noncritical patients infected with SARS-CoV-2, 35% of patients had only mild lymphocytopenia^4^. In contrast, in other two reports with patients with severe disease, the incidences of lymphopenia were 63%^3^ and 70.3%^7^, respectively. Moreover, lymphocytopenia occurred in more than 80% of severe COVID-19 patients^11^. Our results show that the degree of lymphocytopenia may reflect the severity of COIVD-19 disease since the lymphocyte counts were slightly decreased in COVID-19 patients with mild disease, and significantly decreased in patients with severe disease. In addition, our data showed that several immune subsets, such as CD4+ T, CD8+ T, and NK cells, are lower in patients with severity disease.

The reason for patients developing lymphopenia remains unknown. Given that lymphocytes express low level of angiotensin-converting enzyme 2 (ACE2), the cell entry receptor for both SARS-CoV-2 and SARS-CoV^12,16^, and the viral genome is rarely detectable in peripheral blood of SARS-CoV-2 infected patients^5,18^, it is reasonable to speculate that the decrease of peripheral lymphocytes is not directly attributed to SARS-CoV-2 infection. An alternative explanation is that the decease of peripheral lymphocytes is a result of activation-induced apoptosis or aggressive migration from peripheral blood to the lungs, where robust viral replication occurs. Further studies should elucidate the mechanisms responsible for the lymphopenia. It is noteworthy that CD4+ T-cells were profoundly decreased in peripheral blood of severe SARS-COV-2 infected patients. Lessons from HIV infection indicated that a low CD4+ T-cell counts increases the risk of opportunistic infections and lower antiviral immune surveillance, suggesting more attention should to be given to patients in critical condition. Given the limitations of our cross-sectional study, and the clinical significance of lymphopenia and of CD4+ T-cell count, further longitudinal studies with serial sampling are needed to define this further.

The immune response is essential for eradication of the pathogen and resolution of active disease. A recent study demonstrated robust multi-factorial immune responses can be induced by SARS-CoV-2 in a mild case, which is similar to the avian H7N9 disease^21^ and Flavivirus infection^22^. However, the immune and inflammatory response can also be detrimental and cause immune mediated tissue injury, especially in severe cases. It was observed in the 2003 SARS epidemic as well as in the COVID-19 pandemic that pulmonary disease often worsens at 1–2 weeks after onset of respiratory symptoms, coinciding with the onset of virus clearance^17,18^. We recently reported the histology of COVID-19 by obtaining biopsy specimens at autopsy^10,17,23^. Histologic examination revealed diffuse alveolar damage, prominent desquamation of pneumocytes and hyaline membrane formation within left and right lungs with significant interstitial lymphocytic infiltrates. We showed in the histochemical examination of lung tissue obtained from one of our patients with severe disease who died, intense CD4+ and CD8+ T cells infiltrates, with strong granzyme B expression (Fig. 5). Our current study further suggests that the involvement of immune-mediated injury is plausible in the pathogenesis of COVID-19.

In severe patients, despite the absolute counts of CD8+ T cells decreased, these cells showed over-activation, increased T-cell inhibitory molecules expression and increased multiple cytotoxic granules expression, resembling other acute inflammatory processes such as malaria infection, Q-fever, and sepsis^24,25^. Whether these activated cells are antigen-specific or bystander ones warrants further study. However, PD-1 positive T-cells in the peripheral circulation may not only indicate T-cell exhaustion, but could also suggest the presence of antigen-specific T-cells, which needs to be further evaluated^26^. We found that T-cell activation appears to correlate with disease severity in COVID-19 patients as a higher frequency of activated CD8+ T cells (defined by CD38+ and HLA-DR+) occurred in severe patients with longer time after disease onset. Taken together, these findings highlight the involvement of CD8+ T cells for the immunopathogenesis of severe COVID-19. Considering the possibility of clinical application, lymphocyte subset cell counts and activated phenotypes of CD8+ T cells could be used to predict disease outcomes and evaluate new interventions for COVID-19 patients. For example, lymphocyte count is suggested to be closely monitored in COVID-19 patients receiving ECMO^27^.

In conclusion, we provided immunological and inflammatory markers linking disease severity in mild and severe cases of COVID-19 and suggested aberrant activation and dysregulation of CD8+ T cells occur in patients with severe COVID-19 disease which may play an important role in pathogenesis of SARS-CoV-2 infection. Immune-based therapeutic interventions^28^, such as IL-6 receptor antagonist (ChiCTR2000029765) and mesenchymal stem cells (NCT04252118), are undergoing clinical trials. Further studies are required to define immune-mediated tissue injury and identify specific host-directed therapy targets for treatment of the disease.

## Methods

### Ethical approval

This study was approved by the ethics committee of the Fifth Medical Center of PLA General Hospital and written informed consent was obtained from all patients enrolled in this study.

### Patients

Between January 19th and 20th February, a total of 41 patients with laboratory confirmed COVID-19 disease hospitalized at the Fifth Medical Center of PLA General Hospital in Beijing, China, were studied. All patients were diagnosed according to World Health Organization (WHO) interim guidance and were positive for new coronavirus nucleic acid by using SARS-CoV-2 specific RT-PCR test. The patients were classified on basis of disease severity: The mild disease group were defined as with or without pneumonia who were admitted to general wards not requiring intensive care. The severe disease group were those who required critical care and met one or more of these criteria: dyspnea and respiratory rate ≥30/min, blood oxygen saturation ≤93%, PaO_2_/FiO_2_ ratio <300 mmHg, and lung infiltrates on CT scan >50% within 24–48 h, or those who exhibited respiratory failure, septic shock, and/or multiple organ dysfunction/failure. Patient demographics, clinical history, presentations, and findings were recorded. All patients had routine laboratory investigations (full blood count, liver and renal function tests, and coagulation tests).

### Clinical samples

Peripheral venous blood samples were obtained on admission of the COVID-19 patients within 24 h and placed into the vacutainer tube, then centrifuged at 400×*g* for 5 min at 4 °C. The time of sampling relative to the onset of symptoms was recorded. Plasma samples were collected and stored at −80 °C until used. In one patient with severe disease who died, lung tissue samples were obtained post-mortem for immuno-histological analysis.

### Lymphocyte counts and subsets

Absolute lymphocyte counts and subsets were determined using lymphocyte detection kit (Beijing Tongshengshidai Biotechnology Co., LTD, Beijing, China) following the manufacturer’s instructions.

### Flow cytometry

Peripheral blood mononuclear cells (PBMC) were isolated from fresh venous blood using Ficoll density gradient. PBMC samples were stained with the following antibodies: CD3-APC-Cy7 (clone HIT3a), CD3-BV510 (clone OKT3), CD4-BV421 (clone OKT4), CD8-PE-Cy7 (clone SK1), CD45RA-BV510 (clone HI100), CCR7-APC (clone G043H7), CD27-FITC (clone MT271), HLA-DR-FITC (clone L243), CXCR5-BV421 (clone J252D4), PD-1-PE (clone EH12.2H7), CXCR3-BV510 (clone G025H7), CCR4-PerCP-Cy5.5 (clone L291H4), CCR6-PE (clone G034E3), CD25-APC (clone BC96), CD127-FITC (clone A019D5), Perforin-PE-Cy7 (dG9), Granzyme B-AF647 (GB11) were purchased from Biolegend (San Diego, CA); CD4-percp (clone SK3), CD38-APC (clone HIT2), Tim-3-PE (clone 7D3), GNLY-AF488 (clone RB1) were obtained from BD Biosciences (San Diego, CA). Granzyme B, Perforin and GNLY were measured de novo, without prior stimulation with PMA/ionomycin. BD Canto II instrument was used for FACS and the data was analyzed using FlowJo software V10 (Tree star Inc. Ashland, OR).

### Cytokine and chemokine measurement

Plasma levels of 21 different cytokines and chemokines (IL-1β, IL-2, IL-4, IL-5, IL-6, IL-8, IL-10, IL-12P70, IL-17A, IL-17F, IL-22, TNF-α, TNF-β, IFN-γ, IL-1RA, IL-18, G-CSF, RANTES, MCP-1, IP-10, and MIP-1α) in 39 patients infected with SARS-CoV-2 and 24 health controls were determined by flow cytometry using an Aimplex kit (Beijing Quantobio, China) following the manufactures instructions.

### Immunohistochemical staining

Formalin-fixed paraffin-embedded 4-μm sections of lung tissue were subject to immunohistochemistry. Following deparaffinization and rehydration, sections were incubated in 3% H_2_O_2_ in methanol for 30 min at room temperature to block endogenous peroxidase. The sections were then boiled in citrate buffer or EDTA buffer in a microwave oven. The staining was performed using primary antibodies against CD4 (ZSGB-BIO, Beijing, China), CD8 (Abcam, Cambridge, MA), CD68 (ZSGB-BIO), GZMB (Abcam), and incubated at 4 °C overnight. The sections were visualized using the diaminobenzidine solution (DAKO, Carpinteria, CA) and then lightly counterstained with hematoxylin. Images were captured with an inverted fluorescence microscope (PerkinElmer, Norwalk, CT).

### RNAscope

SARS-CoV-2 RNA in formalin-fixed paraffin-embedded (FFPE) tissue were probed by RNAscope reagents, following the manufacture’s protocol. The probes (v-nCoV2019-S, cat. 848561)) targeting SARS-CoV-2 Spike gene and control probes (cat. 320751) are designed by ACD. Briefly, after H_2_O_2_ treatment and protease digestion, FFPE slides were incubated 2 h at 40 °C with probes. The amplifiers and detection solution in the RNAscope 2.5 HD Duplex Reagent kit were added sequentially for hybridization signal amplification for the indicated time. The slides were counterstained with 50% Hematoxylin staining solution for 30 s, and washed with water immediately. Tissue slides were cover slipped with Vectamount Permanent Mounting Medium (Vector Labs, 321584, Burlingame, California). Images were obtained with Perkin Elmer Vectra 3.0 (PerkinElmer).

### Statistical analysis

GraphPad Prism statistical software version 8.0 (GraphPad Software, San Diego, CA) was used. Continuous measurements were displayed as median (IQR) and two group comparisons performed using Mann–Whitney *U*-test. Categorical variables were expressed as count (%) and compared by *χ*² test or Fisher’s exact test between mild and severe group. Pearson correlation test was conducted to assess association between two quantitative variables. *p*-value < 0.05 were considered statistically significant.

### Reporting summary

Further information on research design is available in the Nature Research Reporting Summary linked to this article.

## Supplementary information

Supplementary Information

Peer Review File

Reporting Summary

## Data Availability

The data that support the findings of this study are available from the corresponding author upon reasonable request. Source data are provided with this paper.

## Source data

Source Data
