# Supplementary material for: Immunological and inflammatory profiles in mild and severe cases of COVID-19
Source: Nat Commun. 2020 Jul 8;11:3410. doi: 10.1038/s41467-020-17240-2 (PMC7343781; doi:10.1038/s41467-020-17240-2)
Supplement: Supplementary file 1 — Supplementary Information [file 41467_2020_17240_MOESM1_ESM.pdf]

Supplementary information

Immunological and inflammatory profiles in mild and severe cases of COVID-19

Song *et al.*

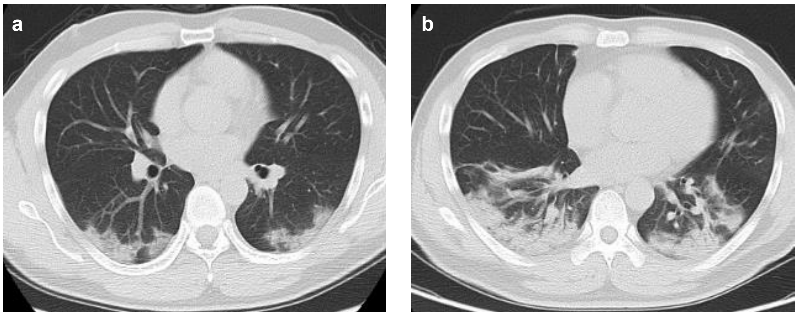

Supplementary Figure 1. Chest computed tomographic images.
Representative computed tomography images from common (**a**) and severe (**b**) patients.

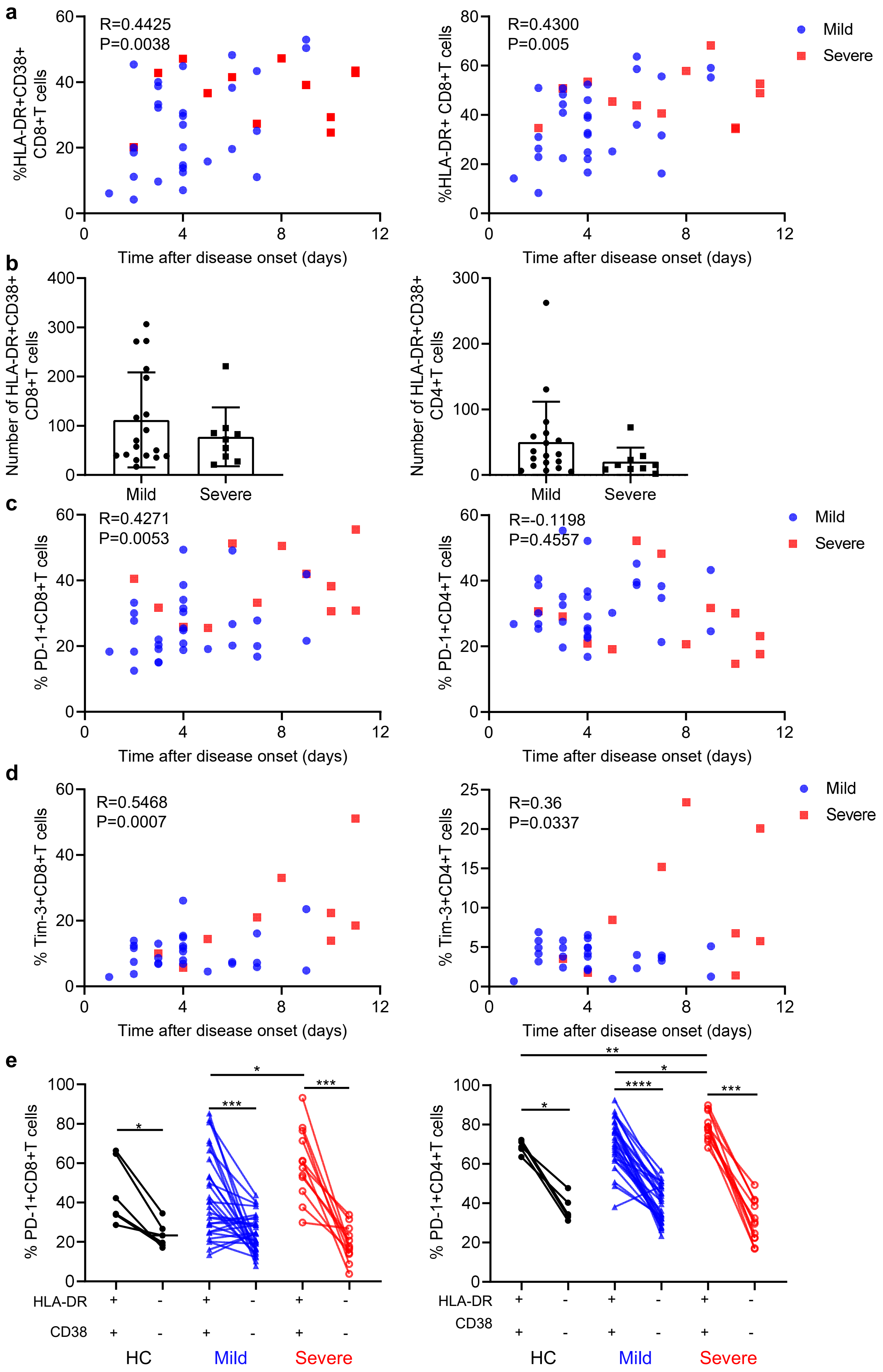

Supplementary Figure 2. Correlation of T cell activation with time after disease onset in COVID-19 patients

(**a**) The association of the frequencies of HLA-DR+CD38+CD8+T (left panel) and HLA-DR+CD8+T cells (right panel) with time after disease onset. (**b**) The number of HLA-DR+CD38+CD8+T and HLA-DR+CD38+CD4+T cells in mild and severe cases. Data are expressed as mean ± SD. (**c-d**) The association of the expression of PD-1 and Tim-3 on CD8+T and CD4+T cells with time after disease onset. (**e**) PD-1 expression on HLA-DR+CD38+CD8+T cells, HLA-DR-CD38-CD8+T cells, HLA-DR+CD38+CD 4+T cells, and HLA-DR-CD38-CD4+T cells in HC, mild and severe cases. Associations were evaluated using Spearman correlations. P value and spearman's rho are presented. Each dot represents a single individual. HC (n=6), Mild (n=29), Severe (n=12). *p < 0.05, ** p < 0.01, *** p < 0.001, ****p < 0.0001, by two-tailed Wilcoxon matched-paired signed rank test or two-tailed Mann-Whitney *U* test for (**e**) (left panel: HLA-DR+CD38+ vs HLA-DR-CD38- (p=0.0313 for HC, p=0.0003 for Mild, and p=0.0005 for Severe), p=0.0149 (mild vs severe); right panel: HLA-DR+CD38+ vs HLA-DR-CD38- (p=0.0313 for HC, p < 0.0001 for Mild, p=0.0005 for Severe), p=0.002 (HC vs Severe) and p=0.0358 (Mild vs Severe)). Source data included as a Source Data file.

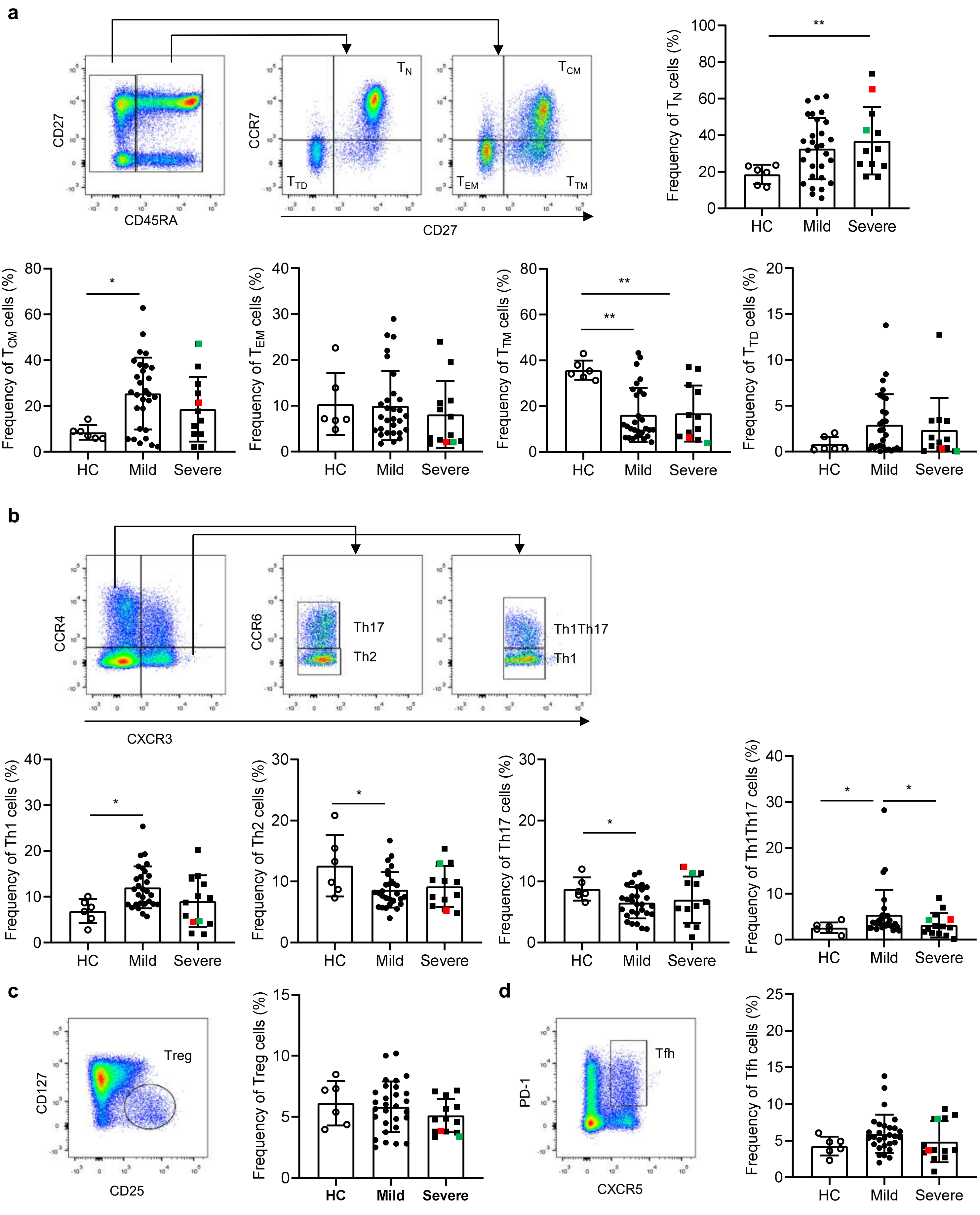

Supplementary Figure 3. SARS-CoV-2 infection altered phenotype of circulating CD4+ T cells

Representative flow cytometry showing the phenotype of CD4+ T cells. (**a**) Memory phenotype of CD4+T cells in PBMC from HC (n=6), mild (n=29) and severe (n=12) SARS-COV-2 infected patients. T_N (naïve: CD45RA+CD27+CCR7+), T_{CM} (central memory: CD45RA-CD27+CCR7+), T_{TM} (transitional memory: CD45RA-CD27+CCR7-), T_{EM} (effector memory: CD45RA-CD27-CCR7-) and T_{TD} (terminal differentiated: CD45RA+CD27-CCR7-). (**b**) Th1, Th2, Th17, and Th1Th17 cells in PBMC from HC (n=6), mild (n=29) and severe (n=12) SARS-COV-2 infected patients. Th1 (CXCR3+CCR4-CCR6-), Th2 (CXCR3-CCR4+CCR6-), Th17 (CXCR3-CCR4+CCR6+), Th1Th17 (CXCR3+CCR4-CCR6+). (**c**) Left dot plot indicated Treg cells gating strategy based on CD25 and CD127 expression, the percentage of Treg cells among HC (n=6), mild (n=29) and severe (n=12) patients was analyzed at the right histogram. (**d**) Left dot plot indicated peripheral Tfh cells gating strategy based on PD-1 and CXCR5 expression, the percentage of Tfh cells among HC (n=6), mild (n=29) and severe (n=12) patients was analyzed at the right histogram. Data are expressed as mean ± SD. *$p < 0.05$, ** $p < 0.01$, by two-tailed Mann-Whitney *U* test for (**a**) ($p=0.0097$ for T_N cells, $p=0.0311$ for T_{CM} cells, $p=0.001$ and $p=0.0069$ for T_{TM}) and (**b**) ($p=0.0077$ for Th1 cells, $p=0.0351$ for Th2 cells, $p=0.0444$ for Th17 cells, and $p=0.0444$ and $p=0.0249$ for Th1Th17 cells). Source data included as a Source Data file.

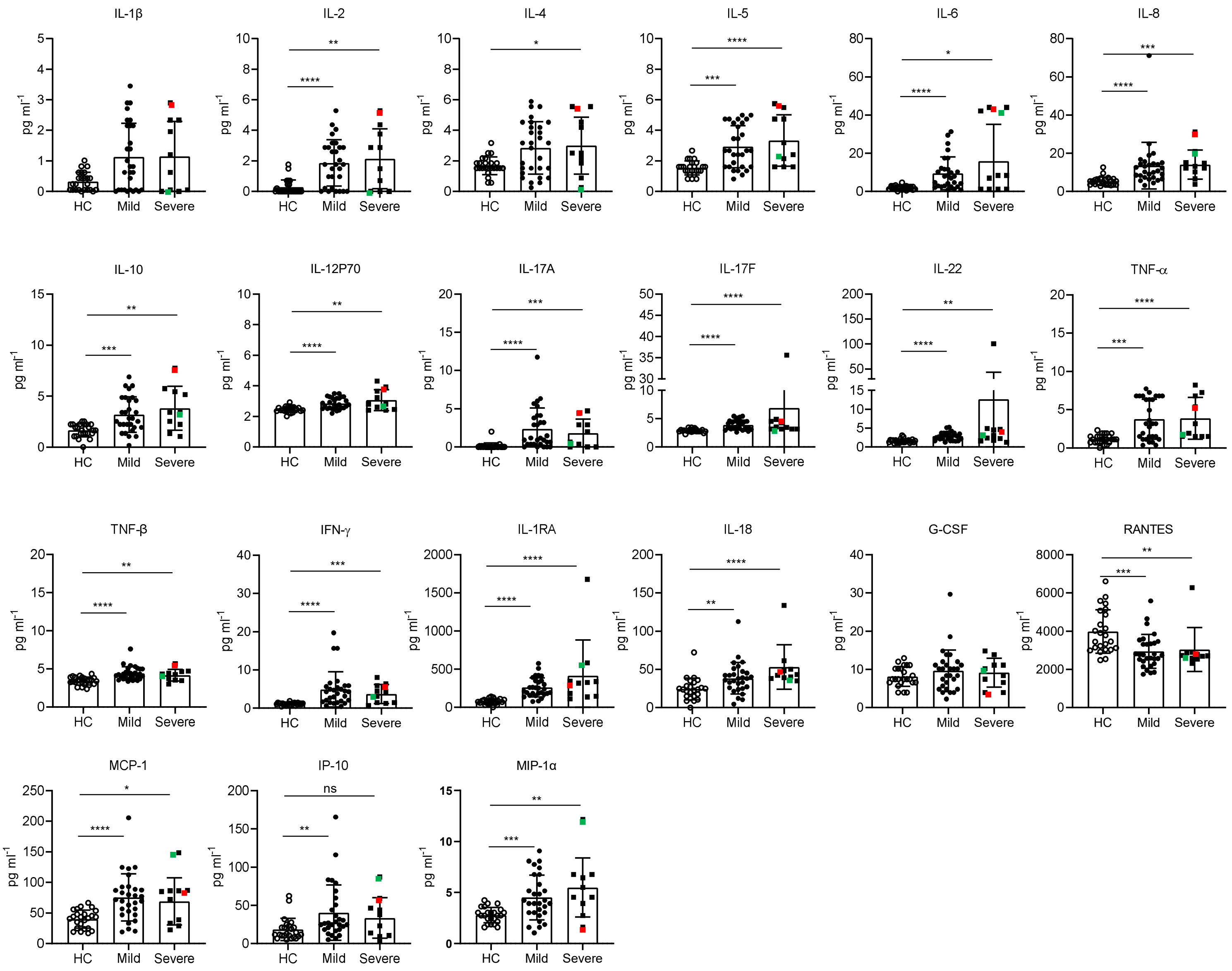

Supplementary Figure 4. Plasma cytokine and chemokine levels of HC, mild and severe patients of SARS-COV-2 infection.
Plasma cytokines and chemokines were determined by flow cytometry using an AIMPLEX kit. HC (n=24), Mild (n=19), Severe (n=10). Each dot represents an individual. Data are expressed as mean ± SD. *p < 0.05, ** p < 0.01, ***p < 0.001, ****p < 0.0001, by two-tailed Mann-Whitney *U* test for IL-2 (p < 0.0001 and p=0.0033), IL-4 (p=0.0224), IL-5 (p < 0.0001 and p < 0.0001), IL-6 (p < 0.0001 and p=0.0325), IL-8 (p < 0.0001 and p=0.0001), IL-10 (p=0.0004 and p=0.0024), IL-12P70 (p < 0.0001 and p=0.0013), IL-17A (p < 0.0001 and p=0.001), IL-17F (p < 0.0001 and p < 0.0001), IL-22 (p < 0.0001 and p=0.0019), TNF-α (p=0.0003 and p < 0.0001), TNF-β (p < 0.0001 and p=0.0052), IFN-γ (p < 0.0001 and p=0.0009), IL-1RA (p < 0.0001 and p < 0.0001), IL-18 (p=0.0017 and p < 0.0001), RANTES (p=0.0006 and p=0.0013), MCP-1 (p < 0.0001 and p=0.0448), IP-10 (p=0.0015) and MIP-1α (p=0.0008 and p=0.0011), respectively. Source data included as a Source Data file.

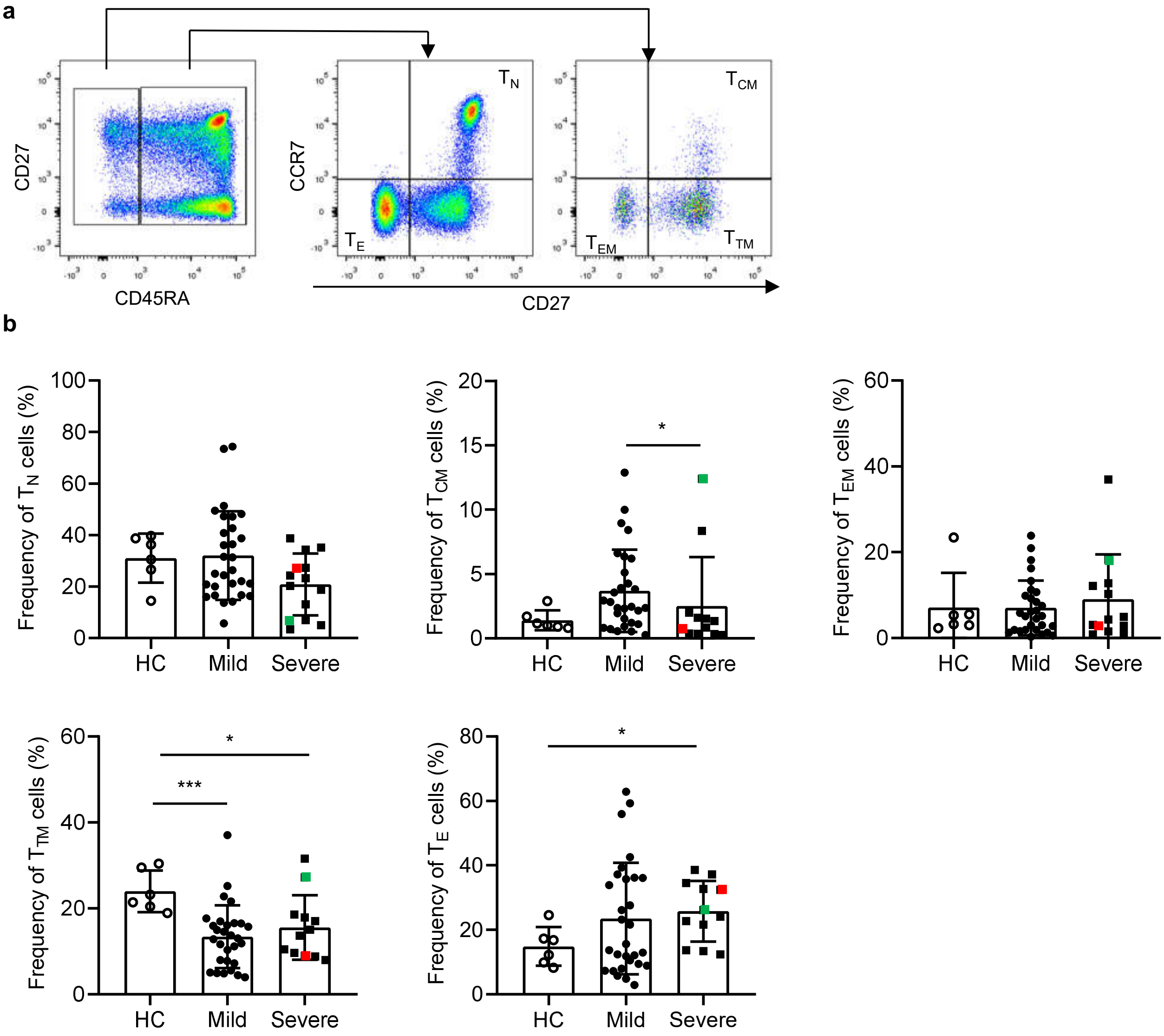

Supplementary Figure 5. SARS-COV-2 infection altered memory phenotype of circulating CD8+ T cells.

(**a**) Dot plots showing that CD8+T cells were divided into distinct subset based on CD45RA, CCR7 and CD27 expression. T_N (naïve: CD45RA+CD27+CCR7+), T_{CM} (central memory: CD45RA-CD27+CCR7+), T_{TM} (transitional memory: CD45RA-CD27+CCR7-), T_{EM} (effector memory: CD45RA-CD27-CCR7-) and T_E (effector: CD45RA+CD27-CCR7-). (**b**) Memory phenotype of circulating CD8+ T cells in PBMC from HC (n=6), mild (n=29) and severe (n=12) SARS-COV-2 infected patients. Data are expressed as mean ± SD. *p < 0.05, *** p < 0.001, by two-tailed Mann-Whitney *U* test for (**b**) (p=0.0365 for T_{CM} cells, p=0.0008 and p=0.0135 for T_{TM} cells, and p=0.0245 for T_E cells). Source data included as a Source Data file.
