# Supplementary material for: Immunological and inflammatory profiles in mild and severe cases of COVID-19
Source: Nat Commun. 2020 Jul 8;11:3410. doi: 10.1038/s41467-020-17240-2 (PMC7343781; doi:10.1038/s41467-020-17240-2)
Supplement: Supplementary file 3 — Reporting Summary [file 41467_2020_17240_MOESM3_ESM.pdf]

nature research

Corresponding author(s): Fu-Sheng Wang
Last updated by author(s): May 25, 2020

Reporting Summary

Nature Research wishes to improve the reproducibility of the work that we publish. This form provides structure for consistency and transparency in reporting. For further information on Nature Research policies, see our Editorial Policies and the Editorial Policy Checklist.

Statistics

For all statistical analyses, confirm that the following items are present in the figure legend, table legend, main text, or Methods section.

n/a	Confirmed	
☐	☒	The exact sample size (*n*) for each experimental group/condition, given as a discrete number and unit of measurement
☐	☒	A statement on whether measurements were taken from distinct samples or whether the same sample was measured repeatedly
☐	☒	The statistical test(s) used AND whether they are one- or two-sided<br>*Only common tests should be described solely by name; describe more complex techniques in the Methods section.*
☒	☐	A description of all covariates tested
☐	☒	A description of any assumptions or corrections, such as tests of normality and adjustment for multiple comparisons
☐	☒	A full description of the statistical parameters including central tendency (e.g. means) or other basic estimates (e.g. regression coefficient) AND variation (e.g. standard deviation) or associated estimates of uncertainty (e.g. confidence intervals)
☐	☒	For null hypothesis testing, the test statistic (e.g. *F*, *t*, *r*) with confidence intervals, effect sizes, degrees of freedom and *P* value noted<br>*Give P values as exact values whenever suitable.*
☒	☐	For Bayesian analysis, information on the choice of priors and Markov chain Monte Carlo settings
☒	☐	For hierarchical and complex designs, identification of the appropriate level for tests and full reporting of outcomes
☒	☐	Estimates of effect sizes (e.g. Cohen's *d*, Pearson's *r*), indicating how they were calculated

Our web collection on statistics for biologists contains articles on many of the points above.

Software and code

Policy information about availability of computer code

Data collection	FACS data were collected on BD Canto II with the BD FACSDiva software (version 6).<br>Images for IHC were collected by Perkin Elmer Vectra 3.0.
Data analysis	The flow cytometry data were analyzed with FlowJo software version 10; All statistical analyses were performed with GraphPad Prism software version 8.

For manuscripts utilizing custom algorithms or software that are central to the research but not yet described in published literature, software must be made available to editors and reviewers. We strongly encourage code deposition in a community repository (e.g. GitHub). See the Nature Research guidelines for submitting code & software for further information.

Data

Policy information about availability of data

All manuscripts must include a data availability statement. This statement should provide the following information, where applicable:
- Accession codes, unique identifiers, or web links for publicly available datasets
- A list of figures that have associated raw data
- A description of any restrictions on data availability

All the generated and analyzed data in the study are included in the Article and its Supplementary Information files. Supplementary information files also include an additional Excel file containing Source Data for all raw data

Field-specific reporting

Please select the one below that is the best fit for your research. If you are not sure, read the appropriate sections before making your selection.

- [x] Life sciences
- [ ] Behavioural & social sciences
- [ ] Ecological, evolutionary & environmental sciences

For a reference copy of the document with all sections, see nature.com/documents/nr-reporting-summary-flat.pdf

Life sciences study design

All studies must disclose on these points even when the disclosure is negative.

Sample size	Forty-one COVID-19 patients were recruited in this study, including 29 mild patients, 12 severe patients. The sample size was determined by the maximum available clinical at the early time point. No prior sample size calculation was performed.
Data exclusions	No data were excluded from the analyses in the study.
Replication	The authors guarantee the findings are reliably reproducible. All the laboratory tests are runned in duplicate.
Randomization	Participants were not chosen randomly, but first 41 patients, who were willing to participate in this study, was enrolled non-selectively during the first wave of COVID-19 in Beijing,
Blinding	All the first 41 patients were enrolled non-selectively and the measurements were performed without prior knowledge of the participant groups.

Reporting for specific materials, systems and methods

We require information from authors about some types of materials, experimental systems and methods used in many studies. Here, indicate whether each material, system or method listed is relevant to your study. If you are not sure if a list item applies to your research, read the appropriate section before selecting a response.

Materials & experimental systems

n/a	Involved in the study	
☐	☒	Antibodies
☒	☐	Eukaryotic cell lines
☒	☐	Palaeontology and archaeology
☒	☐	Animals and other organisms
☐	☒	Human research participants
☒	☐	Clinical data
☒	☐	Dual use research of concern

Methods

n/a	Involved in the study	
☒	☐	ChIP-seq
☐	☒	Flow cytometry
☒	☐	MRI-based neuroimaging

Antibodies

Antibodies used

For flow cytometry
Biolegend antibodies: CD3-APC-Cy7 (clone HIT3a), CD3-BV510 (clone OKT3), CD4-BV421 (clone OKT4), CD8-PE-Cy7 (clone SK1), CD45RA-BV510 (clone HI100), CCR7-APC (clone G043H7), CD27-FITC (clone MT271), HLA-DR-FITC (clone L243), CXCR5-BV421 (clone J252D4), PD-1-PE (clone EH12.2H7), CXCR3-BV510 (clone G025H7), CCR4-PerCP-Cy5.5 (clone L291H4), CCR6-PE (clone G034E3), CD25-APC (clone BC96), CD127-FITC (clone A019D5), Perforin-PE-Cy7 (clone dG9), Granzyme B-AF647 (clone GB11).
BD Biosciences antibodies: CD4-percp (clone SK3), CD38-APC (clone HIT2), Tim-3-PE (clone 7D3), GNLY-AF488 (clone RB1).
For immunohistochemistry
ZSGB-BIO antibodies: CD4 (clone EP204), CD68 (clone KP1)
Abcam antibodies: CD8 (clone C8/144B), GZMB (cat. ab4059)

Validation

Antibody validations were performed by the suppliers per quality assurance literature provided by each supplier. All respective validation data are available on the manufacture`s website.

Human research participants

Policy information about studies involving human research participants

Population characteristics	Between January 19th and 20th February, a total of 41 patients with laboratory confirmed COVID-19 disease hospitalized at the Fifth Medical Center of PLA General Hospital in Beijing was studied, including 29 mild patients and 12 severe patients. The median age was 39 years; 25 (61%) were male. The median time of symptom onset before admission was 5 days. Two severe patients died, and the rest of COVID-19 patients were discharged.
Recruitment	All the available COVID-19 patients at the early time point who were willing to participate in this study were recruited in this study. No self-selection bias existed to the best of our knowledge.
Ethics oversight	This study was approved by the ethics committee of the Fifth Medical Center of PLA General Hospital and written informed consent was obtained from all patients enrolled in this study.

Note that full information on the approval of the study protocol must also be provided in the manuscript.

Flow Cytometry

Plots

Confirm that:

- [x] The axis labels state the marker and fluorochrome used (e.g. CD4-FITC).
- [x] The axis scales are clearly visible. Include numbers along axes only for bottom left plot of group (a 'group' is an analysis of identical markers).
- [x] All plots are contour plots with outliers or pseudocolor plots.
- [x] A numerical value for number of cells or percentage (with statistics) is provided.

Methodology

Sample preparation	Methods, "Flow cytometry" section.
Instrument	BD Canto II
Software	The experiment data were collected by Diva software version 6 (BD Biosciences, USA) and analyzed by FlowJo software version 10 (Tree StarOR).
Cell population abundance	The abundance of relevant cell populations were described on relevant places.
Gating strategy	The gating strategies were shown in each figure where needed.

- [x] Tick this box to confirm that a figure exemplifying the gating strategy is provided in the Supplementary Information.
