# Supplementary material for: Immunological and inflammatory profiles in mild and severe cases of COVID-19
Source: Nat Commun. 2020 Jul 8;11:3410. doi: 10.1038/s41467-020-17240-2 (PMC7343781; doi:10.1038/s41467-020-17240-2)
Supplement: Supplementary file 2 — Peer Review File [file 41467_2020_17240_MOESM2_ESM.pdf]

Editorial Note: This manuscript has been previously reviewed at another journal that is not operating a transparent peer review scheme. This document only contains reviewer comments and rebuttal letters for versions considered at Nature Communications.

REVIEWERS' COMMENTS:

Reviewer #1 (Remarks to the Author):

The authors have addressed my previous concerns

Reviewer #2 (Remarks to the Author):

The manuscript has been greatly improved.

Reviewer #3 (Remarks to the Author):

The authors have addressed the comments by providing additional information on the patients, representative flow cytometry data and also additional clarification and interpretation in the discussion. The manuscript is improved b these modifications.

Reviewer #1:

Remarks to the Author: The authors have addressed my previous concerns

Response: We thank the reviewer again for the positive assessment.

Reviewer #2:

Remarks to the Author: The manuscript has been greatly improved.

Response: We thank the reviewer for the overall positive assessment of our work.

Reviewer #3

Remarks to the Author: The authors have addressed the comments by providing additional information on the patients, representative flow cytometry data and also additional clarification and interpretation in the discussion. The manuscript is improved by these modifications.

Response: We thank the reviewer for the positive comment on our work.
